# A New Method to Reconstruct in 3D the Emission Position of the Prompt Gamma Rays following Proton Beam Irradiation

**DOI:** 10.1038/s41598-019-55349-7

**Published:** 2019-12-11

**Authors:** Costanza M. V. Panaino, Ranald I. Mackay, Karen J. Kirkby, Michael J. Taylor

**Affiliations:** 1 Division of Cancer Sciences, University of Manchester, M13 9PL Manchester, UK; 2 The Christie NHS Foundation Trust, M20 4BX Manchester, UK

**Keywords:** Radiotherapy, Computational science, Experimental particle physics

## Abstract

A new technique for range verification in proton beam therapy has been developed. It is based on the detection of the prompt *γ* rays that are emitted naturally during the delivery of the treatment. A spectrometer comprising 16 LaBr_3_(Ce) detectors in a symmetrical configuration is employed to record the prompt *γ* rays emitted along the proton path. An algorithm has been developed that takes as inputs the LaBr_3_(Ce) detector signals and reconstructs the maximum *γ*-ray intensity peak position, in full 3 dimensions. For a spectrometer radius of 8 cm, which could accommodate a paediatric head and neck case, the prompt *γ*-ray origin can be determined from the width of the detected peak with a *σ* of 4.17 mm for a 180 MeV proton beam impinging a water phantom. For spectrometer radii of 15 and 25 cm to accommodate larger volumes this value increases to 5.65 and 6.36 mm. For a 8 cm radius, with a 5 and 10 mm undershoot, the *σ* is 4.31 and 5.47 mm. These uncertainties are comparable to the range uncertainties incorporated in treatment planning. This work represents the first step towards a new accurate, real-time, 3D range verification device for spot-scanning proton beam therapy.

## Introduction

When compared to conventional x-ray therapy, proton beam therapy (PBT) offers substantial dosimetrical improvements. The depth-dose distribution of proton beams is characterised by a sharp distal fall-off, with the highest amount of energy deposited at the end of the track, in the Bragg peak. This feature is advantageous for cancer treatment: if the beam stops where the target is located, the tumour receives the maximum dose whilst the surrounding healthy tissues are spared^1^. At the moment of writing, many new PBT facilities are in the planning^2^ or construction^3^ stage. One problem that hinders the full exploitation of PBT is the uncertainty in the beam range. Range uncertainty is the uncertainty in the exact position of the distal fall-off of proton beams in biological tissues. Range uncertainty can cause a substantial underdosage of the target, failing the curative intent of the therapy, as well as an overdosage of the adjacent organs-at-risk, leading to unwanted toxicities^4^. In PBT, for non-moving targets, there are several sources of range uncertainty^5^. The most important are: computed tomography (CT) parameters^6–8^, mean ionisation and excitation values^9^ and patient set-up^10^. Most of these uncertainties are initially taken into account in the treatment planning stage, by adding specific margins to the clinical tumour volume (CTV) or through incorporating uncertainty in the treatment planning optimisation, robust optimisation^11^. During fractionated treatments, anatomical changes could also impact the desired dose distribution^12–14^. The most typical anatomical changes are: body weight loss/gain or daily variations in the filling of internal cavities. These changes will be found by imaging during the course of the treatment and may require a plan adaptation. If the dose distribution is not modified in light of severe anatomical changes, the total treatment outcome can be compromised^15^. For this reason, the introduction of range verification during PBT delivery has potential to improve clinical outcomes. Anatomical changes could be detected through daily cone beam CT (CBCT) imaging, however the use of CBCT in the adaptive process for protons is difficult, mainly for the high uncertainty in dose calculation^16^. In contrast, a number of techniques unique to PBT have been proposed in the last decade for real-time range verification. They are based on the detection of the secondary radiation produced naturally during PBT through proton-nuclear inelastic reactions. These techniques provide *in-situ* range verification without any additional burden to the patient^1^.

One proposed method relies on the detection of Prompt-Gamma (PG) rays emitted following proton-nuclear inelastic reactions during therapy. After an inelastic interaction with an incoming proton, the target nucleus can be left in an excited state which can then swiftly return to its ground state via the emission of *γ* rays^4^. These emissions are almost instantaneous, within 10^−9^ s^17^, hence the use of the adjective *prompt* to describe the de-excitation radiation. The PG-ray spectrum is characterised by several discrete *γ*-lines, usually with energies between 2 and 15 MeV. In PBT, only the PG rays emitted by the most abundant isotopes in human tissues, namely carbon (^12^C), oxygen (^16^O) and nitrogen (^15^N), are usually considered^4^. A good correlation between the intensity of the emitted PG rays and the beams end-of-range has been experimentally proven^18^. PG-ray emission occurs along the entire proton track with the maximum intensity located 2–3 millimetres before the Bragg peak; here the cross section for PG-ray production drops as the proton energy decreases^19^. One relevant aspect of PG-ray emission is the production rate: it has been estimated by Verburg *et al*.^18^ that 1.64 · 10^7^ PG rays are emitted per gram of ^16^O per Gray (Gy) of dose delivered in tissue. The delivery of a therapeutic 2 Gy fraction generates a sufficiently high PG-ray yield to allow detection in a clinical environment^20^. An alternative approach is range verification through positron emission tomography (PET) imaging^21^. The clinical adaptation of PG-rays versus PET imaging range verification methods has been compared by Moteabbed *et al*.^20^, finding the former method advantageous.

Since PG imaging was first proposed, several prototypes have been investigated. These prototypes can be divided in collimated, mechanically or electronically, and uncollimated^22^. Mechanical collimated systems are based on a single scintillator, collimated and neutrons-shielded to collect the PG-rays emitted 90° to the beam path^23,24^; they have been initially employed to demonstrate the feasibility of PG-rays detection for range verification. Prototypes with parallel slit collimators, requiring multiple or position-sensitive detectors behind the collimation system, have been suggested^25,26^ afterwards. The concept of a pinhole camera has then been adapted to PG-rays imaging^27^. Subsequently the pinhole opening has been substituted with a single slit of the knife-edge type^28,29^. Knife-edge camera offers an improved spatial resolution and detection efficiency, but allows a one dimensional projection only of the PG-rays along the beam axis^29^. The first two clinical tests of PG-rays imaging for range verification have been performed with knife-edge cameras at Oncoray^30^ and UPenn^31^.

Electronically collimated system are emerging as suitable devices for PG rays as they offer a higher detection efficiency. A Compton camera is a device that determines the energy and the direction of a PG ray as it Compton scatters in the camera’s components. Compton cameras designs comprise different detectors types such as scintillators^32,33^, semiconductors^34,35^ or a combination of them^36–38^. The Electron Tracking Compton Camera (ETCC) is a Compton camera composed of a gaseous time projection chamber, for electron tracking, and a scintillator, for the registration of the scattered photons^39^. Electronically collimation, as opposed to mechanical collimation, allows a three dimensional imaging but suffers from poor geometrical efficiency and low spatial resolution^40^.

Uncollimated systems are based on PG timing, PG peak integral and PG spectroscopy. In PG timing^41^ and PG peak integral^36^ prototypes the width and the peak integral of PG-rays time of flight (TOF) distributions, respectively, are exploited to estimate the proton range. PG Spectroscopy^42^ is based on the identification of the major PG-lines and their intensity. The energy spectra analysis at a single position proximal to the beam range allows an estimation of the target composition and, via the energy dependence on the cross sections, the residual beam range.

The algorithm used to reconstruct the PG-rays distribution is strictly dependent on the prototype. The need to develop complex reconstruction methods has become urgent in Compton cameras. In this context a geometrical line-cone reconstruction has been initially presented by Lojacono *et al*.^43^. Subsequently Maxim *et al*.^44^ showed that the inversion of the Compton transform translates to an analytic filtered backprojection algorithm and developed a reconstruction algorithm that was fast although was unable to deal with complex acquisition designs. Iterative methods, such as the Maximum Likelihood Expectation Maximisation (MLEM)^45,46^ or the origin ensemble algorithm^47,48^, have been subsequently regarded as a more versatile alternative. Reconstructive tools are rapidly evolving; the future of several prototypes for PG-ray detection is based on the algorithms development^22^.

In this article we report the development of a new mathematical reconstruction algorithm to determine the emittance position of ^16^O *γ*-rays naturally produced during PBT. We additionally demonstrate the potential application of this algorithm for range verification.

## Methods

### 3D position reconstruction method

^16^O is one of the most abundant PG-ray emitters in human tissues. The technique developed in this work utilises the 2.741 MeV *γ* emission from the I^*π*^ = 2^−^ state to the I^*π*^ = 3^−^ state in ^16^O followed by the emission of a 6.128 MeV *γ*-ray to the ground state (g.s.). A complete de-excitation decay scheme of ^16^O can be found in Tilley *et al*.^49^. The time difference between the two decays is ~25 ps^49^, which is short compared to the nominal time resolution of scintillator type *γ*-ray spectroscopy detectors (~400–500 ps^50^). Within the limitation of current spectroscopy detector and electronic systems, these two *γ* de-excitations are effectively emitted simultaneously in time and position. The cross section peaks for the reactions ^16^O(p,${{\rm{p}}}_{{\gamma }_{2.742}}^{^{\prime} }$), 2^−^ → 3^−^, and ^16^O(p,${{\rm{p}}}_{{\gamma }_{6.129}}^{^{\prime} }$), 3^−^ → g.s., have been evaluated (Fig. 7 in Kozlovsky *et al*.^17^). Reaction ^16^O(p,${{\rm{p}}}_{{\gamma }_{2.742}}^{^{\prime} }$) has a maximum cross section, ~38 mb, for a proton energy of ~14 MeV while reaction ^16^O(p,${{\rm{p}}}_{{\gamma }_{6.129}}^{^{\prime} }$) has a maximum cross section, ~158 mb, for an energy of ~13 MeV. The average energy, 13.5 MeV, corresponds to a residual proton range of ~2.2 mm in water. Due to the coincidence requirement of the algorithm, the population of the state 2^−^, or above, is essential. For a proton energy of 14 MeV the ^16^O(p,${{\rm{p}}}_{{\gamma }_{2.742}}^{^{\prime} }$)/^16^O(p,${{\rm{p}}}_{{\gamma }_{6.129}}^{^{\prime} }$) cross sections have been compared, with the first being the ~29% of the second. During the proton bombardment of human tissues several 2.741 & 6.128 MeV *γ*-ray *couples* are produced due to ^16^O de-excitation following inelastic nuclear reactions. The simultaneous detection, within the timing resolution of the detection system, coupled with a reconstruction algorithm, allows the identification of the common emission point. The identification uncertainty is proportional to the uncertainties in the position and timing resolutions of the system. The PG-ray distribution has a maximum intensity located a few millimeters proximal to the Bragg peak. For a beam passing through homogeneous tissues with constant oxygen concentration^51^, the beam range can then be determined from the emission points of the detected ^16^O-induced *γ*-ray *couples*.

### Prompt-gamma spectrometer

**Figure 1:**
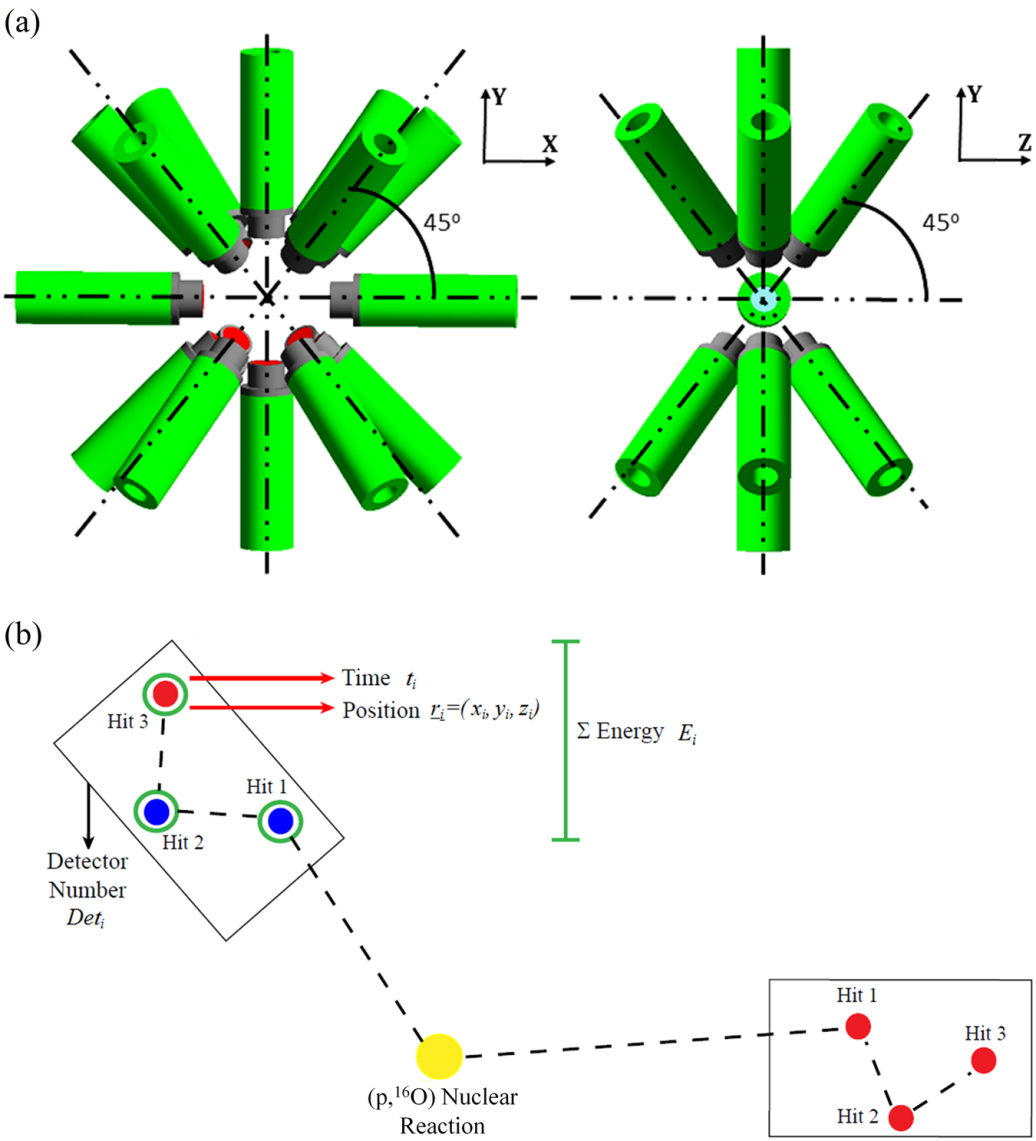
(**a**) The spectrometer under investigation for range verification via PG-ray detection is composed of 16 LaBr_3_(Ce) scintillation detectors arranged in a symmetrical set-up. (**b**) During a simulation, for every PG-ray *γ*_*i*_ recorded in a scintillation module of the spectrometer, several pieces of information are saved: the detector number Det_*i*_, the total energy released E_*i*_, the time t_*i*_, and the coordinates (x_*i*_, y_*i*_, z_*i*_) of the last hit.

To maximise the PG-ray signal, a spectrometer without any mechanical collimation has been designed. As depicted in Fig. 1a, the spectrometer is composed of 16 LaBr_3_(Ce) cylindrical detectors with dimensions 2″ length and 1.5″ diameter. The detectors are arranged as follows: a ring of eight symmetrically-spaced detectors in the vertical plane plus one ring of four detectors at backward angles (45°) and one ring of four detectors at forward angles (45°), with respect to the beam axis. For an isotropic source in the centre of the spectrometer, when the distance between the source and the front face of all detectors is 8 cm, this geometry covers 30% of the total solid angle^52,53^. The energy resolution of LaBr_3_(Ce) (~40 keV FWHM at 1.33 MeV) makes it a suitable detector for high energy PG-ray spectroscopy. In addition, the LaBr_3_(Ce) intrinsic timing resolution is sub-nanosecond from ~keV up to more than 4 MeV, allowing an excellent Time-Of-Flight (TOF) discrimination^54^. Discussions are being held with clinical scientist colleagues for a small design adaptation to enable clinical implementation.

The spectrometer has been modelled using the Geant4 Monte-Carlo Toolkit (version 10.04)^55^. When a *γ*-ray enters the sensitive area of a detector, as shown in Fig. 1b, it interacts a number of times, termed *hits*, before being totally absorbed. For every *γ*_*i*_ detected, several pieces of information are saved:

1. Det_*i*_ = the detector number in which *γ*_*i*_ has been registered;
2. E_*i*_ = the total energy by *γ*_*i*_ in Det_*i*_ deposited (sum of the energy deposited in all hits in Det_*i*_);
3. t_*i*_ = the emission and arrival time difference of *γ*_*i*_ in Det_*i*_;
4. x_*i*_, y_*i*_, z_*i*_ = the coordinates of the last hit of *γ*_*i*_ in Det_*i*_.

For all registered *γ*-rays, this information is available at the end of every simulation.

### MATLAB 3D position reconstruction algorithm

**Figure 2:**
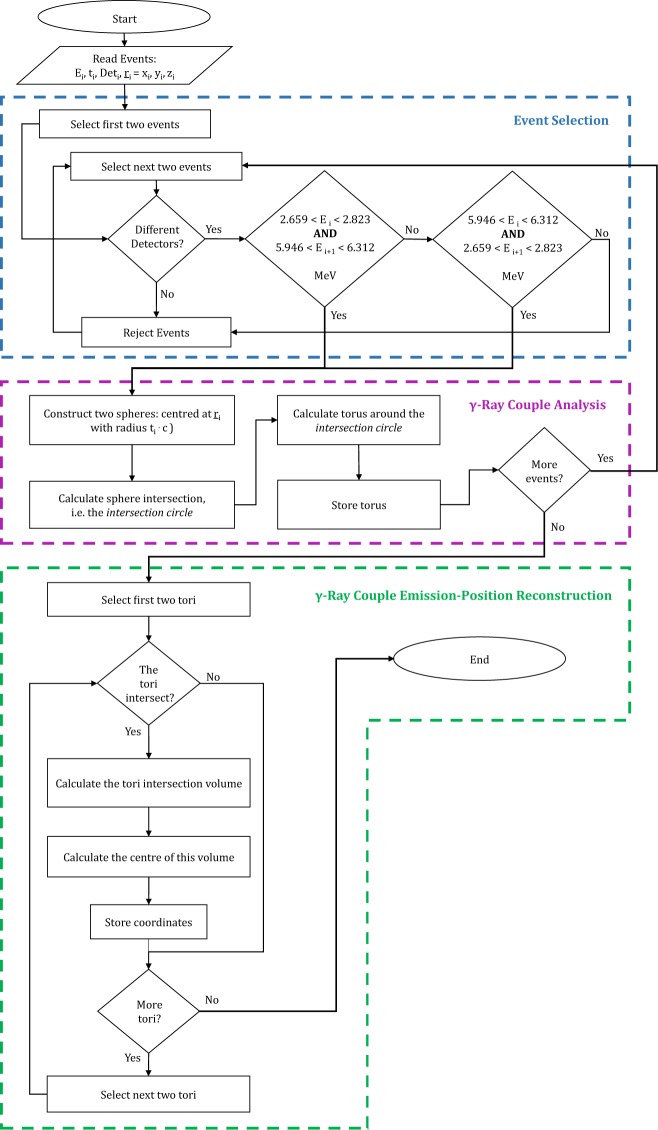
Flowchart of the 3D reconstruction algorithm developed in the MATLAB framework. To reconstruct the PG rays emission-positions, a sequence of steps is undertaken. These steps are represented by three main functions: (1) *γ*-Ray *Couple* Selection, (2) *γ*-Ray *Couple* Analysis, and (3) *γ*-Ray *Couple* Emission-Position Reconstruction. For each PG ray *γ*_*i*_ recorded in the spectrometer, the algorithm requires from the Geant4 simulation the following input data: Det_*i*_, E_*i*_, t_*i*_, and (x_*i*_, y_*i*_, z_*i*_).

In this work, an algorithm has been developed within the MATLAB environment (version R2017b). This algorithm takes as input the detector signals from two coincident *γ* rays and determines their common emission position. In order to reconstruct the emission position, the data goes through three main functions: 1) *γ*-Ray *Couple* Selection, 2) *γ*-Ray *Couple* Analysis, and 3) *γ*-Ray *Couple* Emission-Position Reconstruction. A flowchart detailing the algorithm is shown in Fig. 2 and described in the following sections.

#### Function 1: *γ*-ray couple selection

The algorithm selects *couples* of *γ*-rays *γ*_*i*_ and *γ*_*i*+1_ which satisfy the following criteria:

1. The two events, *γ*_*i*_ and *γ*_*i*+1_, were recorded in coincidence in two different detectors, i.e. Det_*i*_ ≠ Det_*i*+1_.
2. The energies, E_*i*_ and E_*i*+1_, of the two events are 2.741 and 6.128 MeV, irrespective of order.

The energy resolution of a 2″ × 2″ × 8″ LaBr_3_(Ce) crystal has been measured by Dhibar *et al*.^56^ at several photon energies up to 4.433 MeV. Above ~2 MeV the energy resolution is around 3% FWHM (Full Width Half Maximum). The algorithm requires that the energy of one of the two events is in the range 2.659/2.823 MeV while the energy of the other is in the range 5.946/6.321 MeV. These ranges are centred on the two decay energies, namely 2.741 and 6.128 MeV, with the extent reflecting a 3% detector energy resolution. At the end of this function only those events which belong to a *γ*-ray *couple* are saved. Those events which do not fulfil the criteria above are rejected.

#### Function 2: *γ*-ray couple analysis

**Figure 3:**
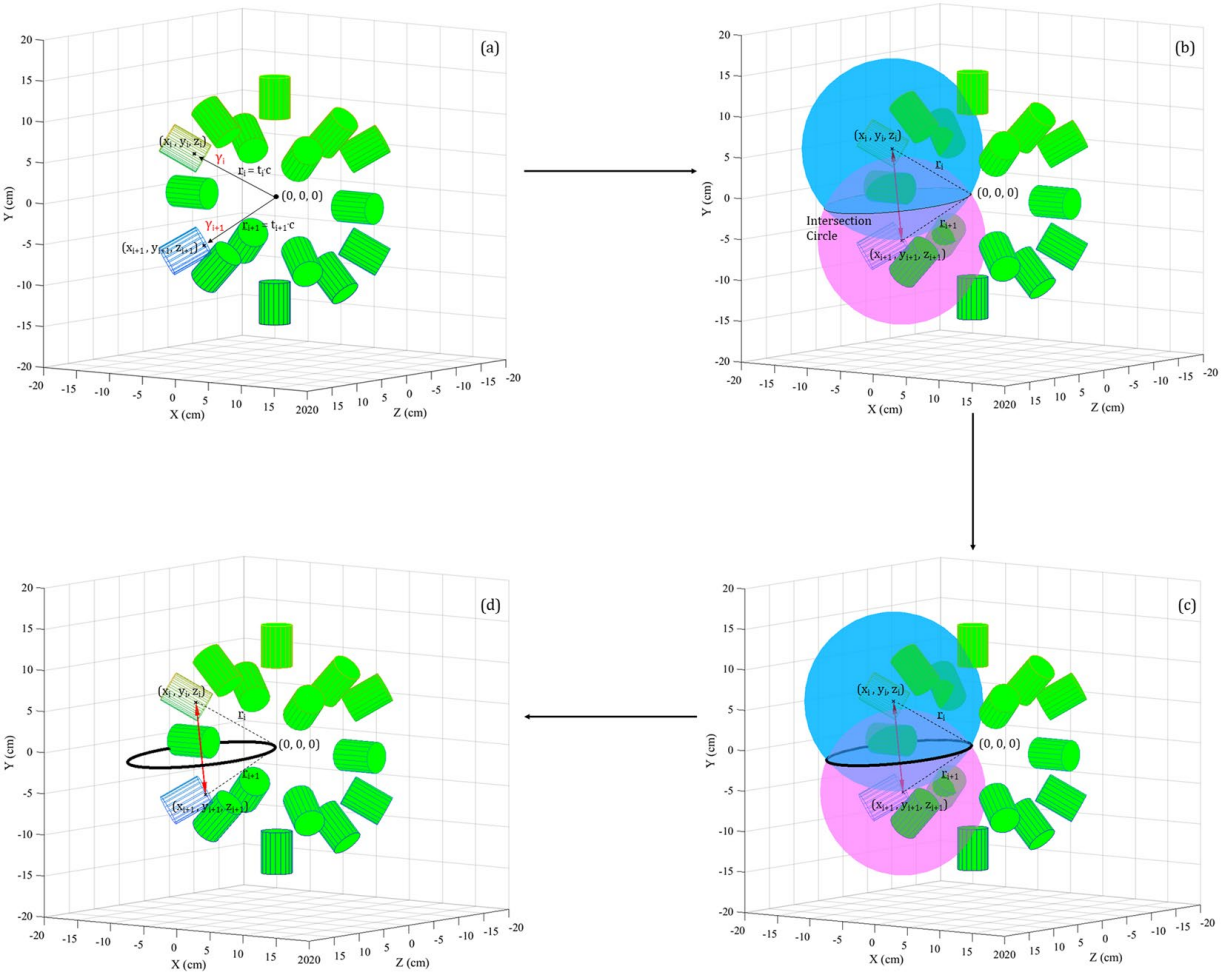
(**a**) In the first function of the algorithm, Event Selection, the input data from Geant4 simulations of those *γ* rays which belong to *couples* are saved. For each event the saved data are: Det_*i*_, E_*i*_, t_*i*_, and (x_*i*_, y_*i*_, z_*i*_). (**b**) In the second function, *γ*-Ray *Couple* Analysis, for each *couple* of *γ* rays *γ*_*i*_ and *γ*_*i*+1_ two spheres are constructed. The hit coordinates (x_*i*_, y_*i*_, z_*i*_) and (x_*i*+1_, y_*i*+1_, z_*i*+1_) represent the centers while the hit times t_*i*_ and t_*i*+1_ are employed to estimate the radii (r_*i*_ = t_*i*_ · c and r_*i*+1_ = t_*i*+1_ · c). The circle which represents the intersection of the two spheres is calculated. (**c**) A torus is constructed around the intersection circle. (**d**) At the end of the second function each previously constructed tours is stored. All the drawings refer to a (p, ^16^O) nuclear reaction in (0, 0, 0).

For each *couple* two spheres are constructed, one for each event in the *couple*. An example of this is illustrated in Fig. 3, for a (p, ^16^O) nuclear reaction at (0, 0, 0), the centre of the spectrometer. As shown in Fig. 3a the centre of each sphere corresponds to the hit coordinates of the associated event while the radius of each sphere is the arrival time of that event multiplied by the speed of light (*c*). The events *γ*_*i*_ and *γ*_*i*+1_, detected in (x_*i*_, y_*i*_, z_*i*_) and (x_*i*+1_, y_*i*+1_, z_*i*+1_), at time t_*i*_ and t_*i*+1_ respectively, are represented by two spheres centred in (x_*i*_, y_*i*_, z_*i*_) and (x_*i*+1_, y_*i*+1_, z_*i*+1_) with radius r_*i*_ = t_*i*_ · c and r_*i*+1_ = t_*i*+1_ · c. As shown in Fig. 3b, the intersection between the two spheres, i.e. an *intersection* circle, is calculated. A torus is constructed around the circle and is stored by the algorithm, Fig. 3c. This geometrical calculation is repeated, resulting in one stored torus per *γ* ray *couple*, Fig. 3d.

The rationale behind the construction of a torus around each *intersection* circle is explained. For every *couple*, the original emission position should lie somewhere on its *intersection* circle. Several small uncertainties, such as the scattering in the detector, affect the spheres parameters. These uncertainties are reflected in the parameters of the circles which, consequently, may not cross each other. In light of this, around every circle, a torus is calculated. For each torus the major radius, i.e. the distance between the centre of the tube and the centre of the torus, corresponds to the radius of the *intersection* circle. For the minor radius, i.e. the radius of the tube, a value of 3 mm was determined from a Monte-Carlo simulation of the *γ*-ray interaction points within the detector medium.

#### Function 3: *γ*-ray *couple* emission-position reconstruction

**Figure 4:**
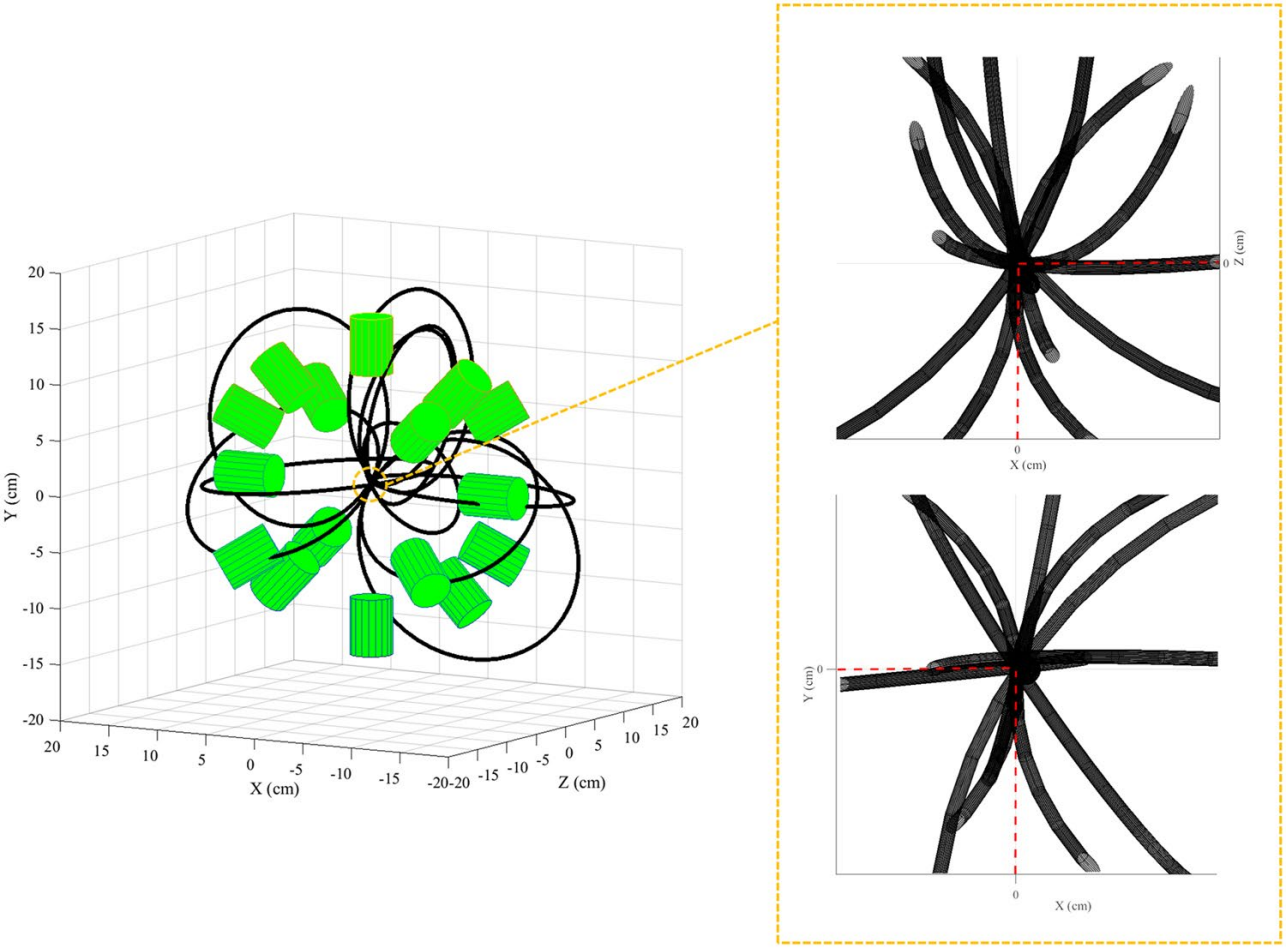
At the end of the second function of the algorithm, *γ*-Ray *Couple* Analysis, *n* tori are stored, where *n* is also the number of all the *γ* rays *couples* originally selected (here reported 11 for display clarity purpose only). A pattern is noticeable: the tori converge to the original position of the (p, ^16^O) nuclear reaction, which, in the present case, is (0, 0, 0).

**Figure 5:**
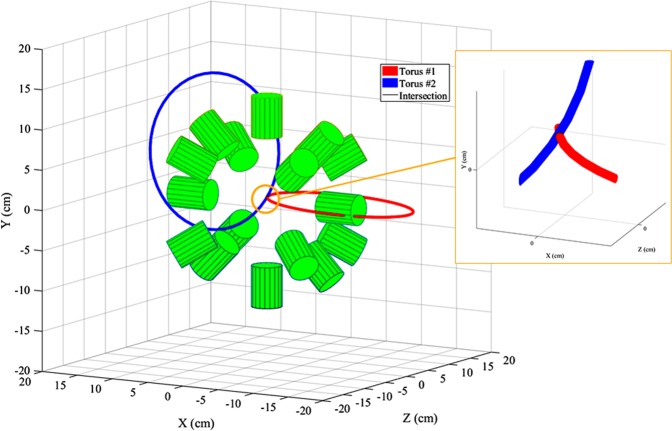
In the third function of the algorithm, *γ*-Ray *Couple* Emission-Position Reconstruction, the intersection between each torus with all the others is calculated. The intersection volume between each chosen *couple* of tori is estimated by triangulating the two tori surfaces and applying a triangle/triangle intersection test routine by Tomas Möller^57^. The centre of each non-null intersection volume is stored as an hypothetical position of the (p, ^16^O) nuclear reaction.

For clarity, only 11 tori are shown in Fig. 4. As highlighted by the inset on the right side of this Figure, the tori converge to the emission position. Each couple of tori is retrieved and, if a non-null volumetric intersection between them exists, the intersection volume is calculated (see Fig. 5). The section of each torus which does not belong to the spectrometer central volume is eliminated before the intersection calculation. This procedure fasten the computational process but allows only the reconstruction of the emitted coordinates located inside the spectrometer. The intersection volume is determined by triangulating the surfaces of the two tori and by applying the triangle/triangle intersection test routine by Tomas Möller^57^. The central point of each intersection volume is calculated and stored as a virtual emission position. The intersection of each torus with all the others (torus n° 1 and torus n°2, …, torus n°1 and torus n°*n*, torus n°2 and torus n°3, …, torus n°2 and torus n°*n*, …, torus n°*n* − *1* and torus n°*n*) is calculated. If *n* is the number of tori and N_*NaN*_ is the number of null intersections between tori, the total number of virtual emission positions N_*emission*–*positions*_ is:

1 $${N}_{emission-positions}=2\cdot n-{N}_{NaN}$$

To obtain the origin of the maximum intensity of the 2.741 & 6.128 MeV ^16^O-induced PG-ray distribution the N_*emission*–*positions*_ x, y, z coordinates of the virtual emission positions are histogrammed.

### Geant4 simulations

The Geant4 Toolkit has been employed to simulate the spectrometer and a clinical 180 MeV proton pencil beam impinging a homogeneous 4 × 4 × 30 cm^3^ water (*G4Water*) phantom. For the LaBr_3_(Ce) detectors, an energy resolution of 3% FWHM^58^ and a time resolution of 280 ps^59^ have been chosen. For the spectrometer, to subtend a high solid angle with respect to the PG-ray emitted in proximity of the Bragg peak and to obtain meaningful results within a reasonable computational time, the internal radius was set to 8 cm.

**Figure 6:**
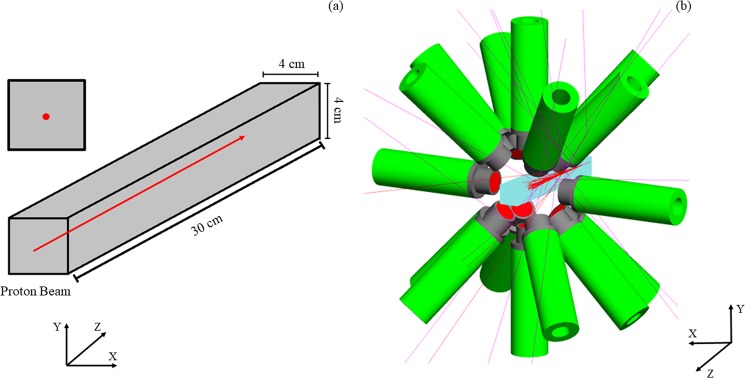
Geometry of the Geant4 simulation. (**a**) A 4 × 4 × 30 cm^3^ water phantom is irradiated by a 180 MeV clinical proton pencil beam. The beam direction coincides with the phantom central axis (Z axis). (**b**) Both the phantom and the beam are modelled in the centre of the spectrometer. The water phantom has been modelled so that the Bragg peak depth for the 180 MeV beam corresponds to the centre of the spectrometer, i.e. the point (0, 0, 0). This as to ensure that the ^16^O-induced PG rays emitted close to the Bragg peak are detected by the spectrometer with the maximum solid angle (Ω = 30%).

As shown in Fig. 6, both the beam and the phantom have been modelled in the central area of the spectrometer. The beam direction coincides with the phantom central axis (Z axis). The water phantom has been modelled so that the Bragg peak depth for the 180 MeV beam corresponds to the centre of the spectrometer. This is to ensure that the PG rays emitted close to the Bragg peak are detected by the spectrometer with the maximum solid angle. The proton energy distribution was set as Gaussian with a sigma of 1 MeV. The sigma value for the lateral spread was set as 4 mm. These parameters were chosen as they represent typical values determined on our system.

The number of initial protons simulated was 10^8^. The PG rays, emitted in the (p, ^16^O) nuclear reactions, have been recorded by the spectrometer. The simulation outputs have been processed with the algorithm to reconstruct, in full 3 dimensions, the beam end-of-range value in the phantom. In addition, a scoring mesh (20 × 20 × 150 bins), with the same size and position of the phantom was created. The quantities scored by this mesh were: 1) the energy deposition per voxel and 2) the 2.741 & 6.128 MeV ^16^O-induced PG-ray distribution. These quantities are used as a benchmark for the reconstruction algorithm results.

To model the interactions in the Geant4 simulation both electromagnetic (*EmStandardPhysics*-*Option*4 *EmExtraPhysics*) and hadronic (*HadronElasticPhysics*, *HadronPhysicsQGS* – *BIC*, *IonBinaryCascadePhysics*, *NeutronTrackingCut* and *StoppingPhysics*) physics lists have been combined together. The *IonBinaryCascadePhysics* was selected as it has been proved^60,61^ that this physics list is the most suitable to model the PG-ray emission. For all particles, the cut has been set to 0.5 mm.

## Results

**Figure 7:**
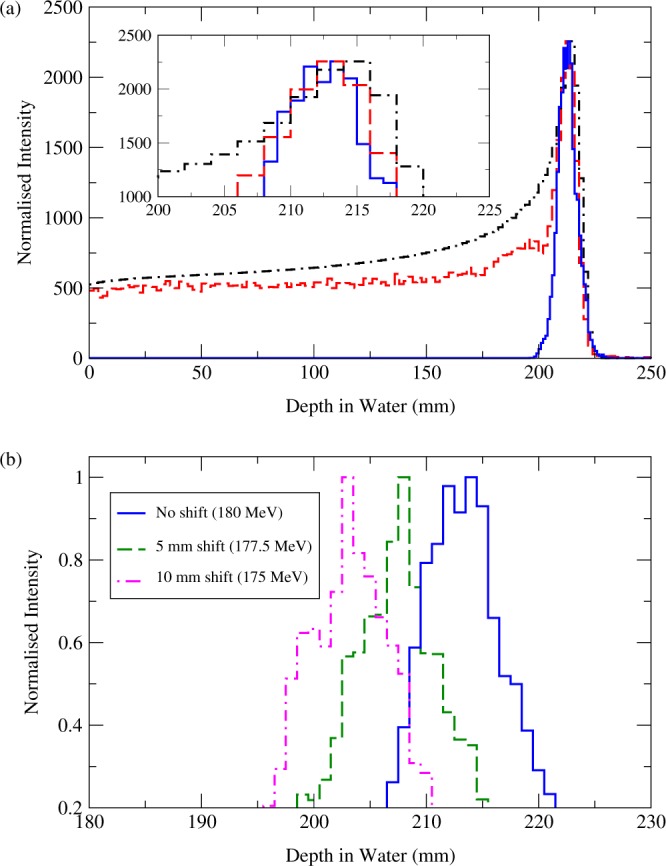
(**a**) A clinical 180 MeV proton pencil beam impinges a 4 × 4 × 30 cm^3^ water phantom. Two quantities are scored by the phantom: the proton energy deposition (*black* dot-dashed curve) and the 2.741 & 6.128 MeV ^16^O-induced PG rays (*red* dashed curve). In addition the maximum intensity emission origin of the 2.741 & 6.128 MeV ^16^O-induced PG rays, detected with the spectrometer and reconstructed with the algorithm, is plotted (*blue* curve). (**b**) Two clinical proton pencil beams, with energy 175 and 177.5 MeV, impinge the same water phantom. The phantom is modelled so that the spectrometer centre coincides with the Bragg peak for a 180 MeV beam. This translates into a range undershoot of 5 and 10 mm, respectively. The maximum intensity emission origin of the 2.741 & 6.128 MeV ^16^O-induced PG rays, detected with the spectrometer and reconstructed with the algorithm, is plotted for the 175 (*purple* dot-dashed curve) and 177.5 MeV (*green* dashed curve). For comparison the same plot is shown (*blue* curve) for a 180 MeV beam. All distributions are along the Z axis.

In Fig. 7a, the result of a simulation with a 180 MeV proton pencil beam impinging a water phantom is illustrated. This Figure shows the profiles, along the Z axis, of the following normalised distributions: the proton energy deposition as scored by the mesh (*black* dot-dashed curve), the 2.741 & 6.128 MeV ^16^O-induced PG-ray distribution also scored by the mesh (*red* dashed curve), and the origin of the maximum intensity of the 2.741 & 6.128 MeV ^16^O-induced PG rays as determined by the algorithm (*blue* curve). The total number of 2.741 & 6.128 MeV ^16^O-induced PG-rays *couples*, selected by the algorithm in Function 1, is 826. The two mesh-based distributions refer to a phantom with 2 × 2 × 2 mm^3^ voxels. Conversely, for the algorithm-reconstructed distribution, the phantom has been divided in 1 × 1 × 1 mm^3^ voxels. As the mesh scored quantities are used solely for benchmarking a larger voxel size was chosen to reduce computation time. In the mesh scored distributions the Bragg peak position and the maximum intensity position of the 2.741 & 6.128 MeV ^16^O-induced PG rays are located at a depth of 21.60 and 21.40 cm, respectively. By applying a Gaussian fit on the algorithm reconstructed data the maximum intensity position of the 2.741 & 6.128 MeV ^16^O-induced PG rays is at a depth of 21.37 ± 0.42 cm. No smoothing is applied prior to the fit, however the histogram binning may have an effect.

For a clinical implementation of the system, a spectrometer internal radius of 8 cm appears to be only suitable for a very small treatment volume. The most likely clinical scenario for this radius could be a paediatric head and neck case. Additional simulations have been performed to investigate the performance of the spectrometer in different clinical scenarios. In all simulations, the beam and the water phantom have been modelled in the central area of the spectrometer as described in Section 2.4. The number of initial protons simulated has been kept fixed at 10^8^. The spectrometer internal radius has been set to 15 and 25 cm to represent, respectively, an adult head and neck (Fig. S1) and a thoracic treatment (Fig. S2). With respect to the configuration previously described, the solid angle subtended by the spectrometer with respect to the origin (0, 0, 0) decreases from 30% for a 8 cm radius to 9% for a 15 cm radius and to 3% for a 25 cm radius. The total number of 2.741 & 6.128 MeV ^16^O-induced PG-ray *couples*, selected by the algorithm in Function 1, is 387 and 191 when the radius is 15 and 25 cm, respectively. The variation of the spectrometer performance with the internal radius is shown in Table 1. Both the lateral spread (standard deviation), *σ*, and the centroid, *μ*, of the algorithm-reconstructed maximum intensity ^16^O PG-ray distribution are reported for each chosen radius. These values have been obtained by applying a Gaussian fit to the reconstruction data.

**Table 1:** Evaluation of the system performance, in reconstructing the end-of-range position of a proton pencil beam, by varying the spectrometer internal radius and the beam energy.

Spectrometer Internal Radius Variation			
*Spectrometer Radius (cm)*	*Hypothetical Treatment Site*	*Centroid μ (cm)*	*Standard Deviation σ (mm)*
8	head and neck (paediatric)	21.37	4.17
15	head and neck (adult)	21.41	5.65
25	thorax (adult)	21.45	6.36
**Proton Beam Energy Variation**			
***Proton Beam Energy**** (****MeV****)*	|***Shift***| *(****mm****)*	***Centroid μ**** (****cm****)*	***Standard Deviation σ**** (****mm****)*
180	0	21.37	4.17
177.5	5	20.84	4.31
175	10	20.31	5.47

The lateral spread (standard deviation), *σ*, and the centroid, *μ*, of the algorithm-reconstructed ^16^O PG-ray distribution, as obtained through the application of a Gaussian fit, are reported. As a reference, the results of a simulation with 8 cm spectrometer internal radius and 180 MeV beam energy are shown when both variations are performed. When different spectrometer internal radii (8, 15, and 25 cm), representing different hypothetical treatment sites, are modelled, the beam energy has been always kept at 180 MeV. Conversely, when the Bragg peak is shifted, with respect to the centre of the spectrometer, by 5 and 10 mm, the spectrometer internal radius has always been set to 8 cm.

Simulations have been performed to test the spectrometer ability to estimate range deviations from a peak position expected at (0, 0, 0). With respect to the previous analysis the beam energy has been decreased to 177.5 and 175 MeV, which corresponds, to a peak depth, in water, of 21.06 and 20.54 cm and to a range shift of ~5 and ~10 mm relative to the origin (0, 0, 0). In both simulations the number of initial protons was 10^8^ and the spectrometer internal radius was 8 cm. The total number of *couples* is 806 and 766, when the shift is 5 and 10 mm, respectively. Fig. 7b depicts, along the Z axis, the maximum intensity emission origin of the 2.741 & 6.128 MeV ^16^O-induced PG ray, detected by the spectrometer and reconstructed by the algorithm, when the beam energy is 180 (*blue* curve), 177.5 (*green* dashed curve), and 175 MeV (*purple* dot-dashed curve). For the three energies, a Gaussian fit is applied to the algorithm reconstructed data. The lateral spread, *σ*, and the centroid, *μ*, obtained through the fit, are reported in Table 1.

## Discussion

An excellent correlation is observed in Fig. 7a between the two mesh-scored distributions: the 2.741 & 6.128 ^16^O-induced PG rays (*red* dashed curve) and the energy deposition due to the electronic stopping of the proton beam (*black* dot-dashed curve). This is consistent with the results of previous in silico studies^23,62^ and with the outcomes of measurements by Verburg *et al*.^18^. The 2 mm shift between the depth of the Bragg peak and the depth at which the PG rays are emitted with maximum intensity, highlighted in the inset in the same Figure, is due to the cross-section for ^16^O PG-ray emission. As shown is Section 2.1 the total PG cross section for ^16^O maximises for incident protons of ~14 MeV. The distribution (*blue* curve) of *γ*-ray emittance positions, reconstructed by the algorithm along the Z axis (beam direction), is in agreement with the maximum intensity of the ^16^O PG-ray distribution.

Table 1 shows the results of an investigation into the spectrometer and algorithm performance for increasing treatment volume. To use the device for adult head and neck or adult thoracic based tumours the spectrometer internal radius would have to be set to a value greater than 8 cm. The reconstruction algorithm takes as one of its inputs the *γ*-ray detection time, therefore the relative accuracy of the time-of-flight determination increases with flight path, i.e. source to detector distance, up to a limit fixed by the timing resolution of the system. Conversely, for a fixed number of protons/*γ*-rays, the geometrical efficiency of the spectrometer decreases with increasing radius. For a spectrometer radius of 8, 15, and 25 cm, assuming a proton beam current of 2 nA^63^, the estimated count rate per detector is 21, 7.8, and 3 Mcps, respectively. At the rate for a realistic treatment radius of 25 cm, using 250 MHz digital electronics, pulse pile up should not be a significant problem. For smaller radii and increased count rate the use of digital electronics would allow logic pile-up rejection or pile-up recovery through pulse shape analysis. The results of an investigation into the spectrometer and algorithm performance for a range undershoot of 5 and 10 mm are presented in Table 1 and graphically depicted in Fig. 7b. Due to the symmetry of the spectrometer these results reflects shifts caused by a range overshoot of the same magnitude.

This work uses a computationally reasonable number of initial protons (10^8^), which is comparable to the number of protons delivered in a pencil beam spot. At 68% confidence level, the reconstruction uncertainty is below 7 and 6 mm, for the 25 cm radius case and the 8 cm radius case with a range undershoot of 10 mm, respectively. These uncertainties are comparable to the ones typically fed in to robust planning or the usual margins imposed in PBT planning. Following the recipe of Massachusetts General Hospital (MGH), 3.5% of the range plus 1 mm^5^, for a 180 MeV clinical beam the usual margin is 5.7 mm at 68% confidence level. Currently, the reconstruction obtained in the present work is comparable with the performances of the prototypes based on the Compton camera technique^64^. In a second test on patients, Xie *et al*.^31^, using the IBA knife-edge prototype, estimated the shift of the Bragg peak position relative to the plan, with a ±2 mm precision. Hueso-Gonzalez *et al*.^42^ claims that, with the PG spectroscopy system developed in MGH perfectly aligned on the couch, the absolute range can be reconstructed, in ideal experimental phantoms, with a mean precision of 1.1 mm at 95% confidence level.

For a 180 MeV proton beam, when the internal spectrometer radius is 8 cm, the total number of *γ*-rays detected is 5,591,199. Amongst these events the 1.34% of them have energies in the two ranges discussed in Section 2.3.1. The number of events accepted by the algorithm in Function 1 is 1,652 (826 *couples*). The authors are additionally investigating the possibility of including, as acceptance criteria, those events whose energy belongs to the single/double escape peaks. With this variation, for the 8 cm radius case, the number of couples rises to 3,884, a ~5 fold increase.

The spectrometer has been modelled with realistic energy and temporal resolution. The detectors of choice for this work are large crystal LaBr_3_(Ce) scintillators. These crystals possess internal activity, predominantly due to the decay of ^138^La. The energy of the ^138^La *γ*-rays does not overlap with the ^16^O PG rays of interest. In addition, the coincidence requirement of the algorithm rejects the activity of these *γ*-rays. The rate of the LaBr_3_(Ce) internal activity was measured to be 0.85 cts/(s/cm^3^) in the energy interval 70–5000 keV^54^, slow compared to the (p, ^16^O) reaction rate^49^. For all these reasons the LaBr_3_(Ce) internal activity has not been modelled. Additionally, due to the coincidence requirement in the algorithm. the neutrons-induced *γ* rays are rejected from the reconstruction process.

The accuracy of this technique is influenced by two main factors, the *γ*-ray interaction position (x_*i*_, y_*i*_, z_*i*_) in the detector medium and its flight time (t_*i*_). Monte-Carlo simulations can produce detector data with exact final *γ*-ray interaction positions, however, in reality this hit position is not known to the same precision. Running the simulations many times generates a mean distribution of hits for each detector. A probability density function is then derived from this distribution and sampled to generate interaction co-ordinates needed by the algorithm for non position sensitive detectors. The employment of segmented detector modules, with improved position resolution, is under evaluation.

Similarly the exact time difference between *γ*-ray emission and detection, in reality, is also not available. A common start time provided by a suitable timing device could be employed. If this is achieved, the hit times *t*_*i*_ and *t*_*i*+1_ of the two events *i* and *i* + *1* can be individually inferred. In this case the developed algorithm would not need any modification to determine the beam range. An alternative algorithm is under development; it can reconstruct the *γ*-ray origin without the need for a start time and only needs the *γ* ray detector arrival times as input.

All the reconstruction results presented in this study were obtained within 30 minutes (Windows 10 64-bit with Intel Core i7-6700 @ 3.41 GHz CPU and 16 GB RAM). The reconstruction algorithm currently runs in a MATLAB environment and a significant decrease in this computational time could be achieved by porting this to a pre-compile binary via a high-level language such as C or C++. Further improvements could be made by porting the algorithm to hardware and both of these options are currently being explored.

## Conclusions

A new technique for range verification in proton beam therapy has been developed. It is based on the detection of the prompt *γ* rays that are emitted naturally during delivery. A spectrometer comprising 16 LaBr_3_(Ce) detectors in a symmetrical configuration is employed to record the prompt *γ* rays emitted along the proton path. An algorithm has been also developed that takes as inputs the LaBr_3_(Ce) detector signals and reconstructs the maximum intensity peak position, in full 3 dimensions. The ability to determine proton range in 3D is well suited for spot-scanning systems and for detecting non-uniform anatomical changes such as tumour shrinkage. The spectrometer-algorithm performance has been first investigated for a mono-energetic 180 MeV clinical beam with varying spectrometer radii. The results show that accommodating an adult patient (25 cm spectrometer radius) the proton range could be determined with an uncertainty below 7 mm at 68% confidence level. Additional simulations have been performed with a shift between the beam range and the system origin. In case of a 10 mm range undershoot the PG-ray emission position is reconstructed with an uncertainty below 6 mm at 68% confidence level. Further developments are ongoing to reach the ultimate goal of a clinically compliant system for on-line, real-time range verification.

## Supplementary information

Supplementary Information

## Data Availability

The datasets generated during and/or analysed during the current study are available from the corresponding author on reasonable request.
