# Supplementary material for: A New Method to Reconstruct in 3D the Emission Position of the Prompt Gamma Rays following Proton Beam Irradiation
Source: Sci Rep. 2019 Dec 11;9:18820. doi: 10.1038/s41598-019-55349-7 (PMC6906450; doi:10.1038/s41598-019-55349-7)
Supplement: Supplementary file 1 — Supplementary Information [file 41598_2019_55349_MOESM1_ESM.pdf]

A New Method to Reconstruct in 3D the Emission Position of the Prompt Gamma Rays following Proton Beam Irradiation

Costanza M. V. Panaino[1,2,*], Ranald I. Mackay[1,2], Karen J. Kirkby[1,2], and Michael J. Taylor[1,2]

[1]Division of Cancer Sciences, University of Manchester, M13 9PL, Manchester, UK
[2]The Christie NHS Foundation Trust, M20 4BX, Manchester, UK
[*]corresponding author costanza.panaino@postgrad.manchester.ac.uk

ABSTRACT

A new technique for range verification in proton beam therapy has been developed. It is based on the detection of the prompt γ rays that are emitted naturally during the delivery of the treatment. A spectrometer comprising 16 $LaBr_3$(Ce) detectors in a symmetrical configuration is employed to record the prompt γ rays emitted along the proton path. An algorithm has been developed that takes as inputs the $LaBr_3$(Ce) detector signals and reconstructs the maximum γ-ray intensity peak position, in full 3 dimensions. For a spectrometer radius of 8 cm, which could accommodate a paediatric head and neck case, the prompt γ-ray origin can be determined from the width of the detected peak with a σ of 4.17 mm for a 180 MeV proton beam impinging a water phantom. For spectrometer radii of 15 and 25 cm to accommodate larger volumes this value increases to 5.65 and 6.36 mm. For a 8 cm radius, with a a 5 and 10 mm undershoot, the σ is 4.31 and 5.47 mm. These uncertainties are comparable to the range uncertainties incorporated in treatment planning. This work represents the first step towards a new accurate, real-time, 3D range verification device for spot-scanning proton beam therapy.

SUPPLEMENTARY INFORMATION

Variation of the Spectrometer Internal Radius

Two simulations have been performed in Geant4; a water phantom (4x4x30 cm^3) is hit by a clinical 180 MeV proton pencil beam. Both the phantom and the beam are in the central area of the spectrometer and the beam direction coincides with the phantom central axis (Z axis). The phantom has been modelled so that the Bragg peak depth for the 180 MeV beam corresponds to the centre of the spectrometer. This is to ensure that the PG rays emitted close to the Bragg peak are detected by the spectrometer with the maximum solid angle. The number of initial protons simulated has been kept fixed at 10^8. The spectrometer internal radius has been set at 15 and 25 cm. The radius has been varied to represent different clinical scenarios: a head and neck treatment (Fig. S1) and a thoracic treatment (Fig. S2). In other words the solid angle subtended by the spectrometer with respect to the origin (0, 0, 0) decreases to 9% (radius 15 cm) and to 3% (radius 25 cm).

The PG rays, emitted in the (p, ^{16}O) nuclear reactions, have been recorded by the spectrometer. The simulations outputs have been processed with the algorithm to reconstruct, in full 3 dimensions, the beam end-of-range value in the phantom. In addition, a scoring mesh (20x20x150 bins), with the same size and position of the phantom, has been implemented. The quantities scored in the mesh were: 1) the energy deposition per voxel and 2) the 2.741 & 6.128 MeV ^{16}O-induced PG-ray distribution. These quantities are used as a benchmark for the reconstruction algorithm results. The two mesh-based distribution refer to a phantom with 2x2x2 cm^3 voxels. Conversely, for the algorithm-reconstructed distribution, the phantom has been divided in 1x1x1 cm^3 voxels. As the mesh scored quantities are used solely for benchmarking, a larger voxel size was chosen to reduce computation time.

The total number of 2.741 & 6.128 MeV ^{16}O-induced PG-ray *couples*, selected by the algorithm in Function 1, is 387 and 191, when the radius is 15 and 25 cm, respectively. If the *couples* detection rate is Poissonian then the signal-to-noise ratio, SNR, at 191 *couples*, is $\sim$ 14.

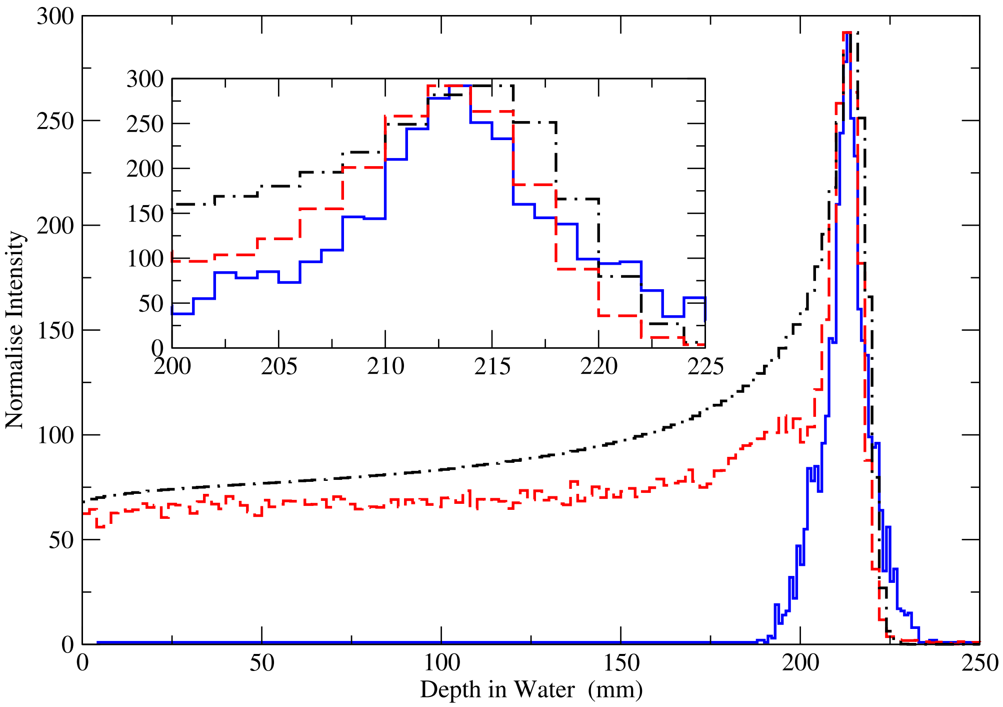

Figure S1. A clinical 180 MeV proton pencil beam impinges a water phantom. Two quantities are scored by the phantom: the proton energy deposition (*black* curve) and the 2.741 & 6.128 MeV ^{16}O-induced PG-ray (*red* curve). In addition addition the maximum intensity emission origin of the 2.741 & 6.128 MeV ^{16}O-induced PG-ray, detected with the spectrometer and reconstructed with the algorithm, is plotted (*blue* curve). Distributions are shown along the Z axis. The internal radius of the spectrometer is 15 cm (solid angle $\Omega = 9\%$ subtended by the spectrometer at the central point (0, 0, 0)).

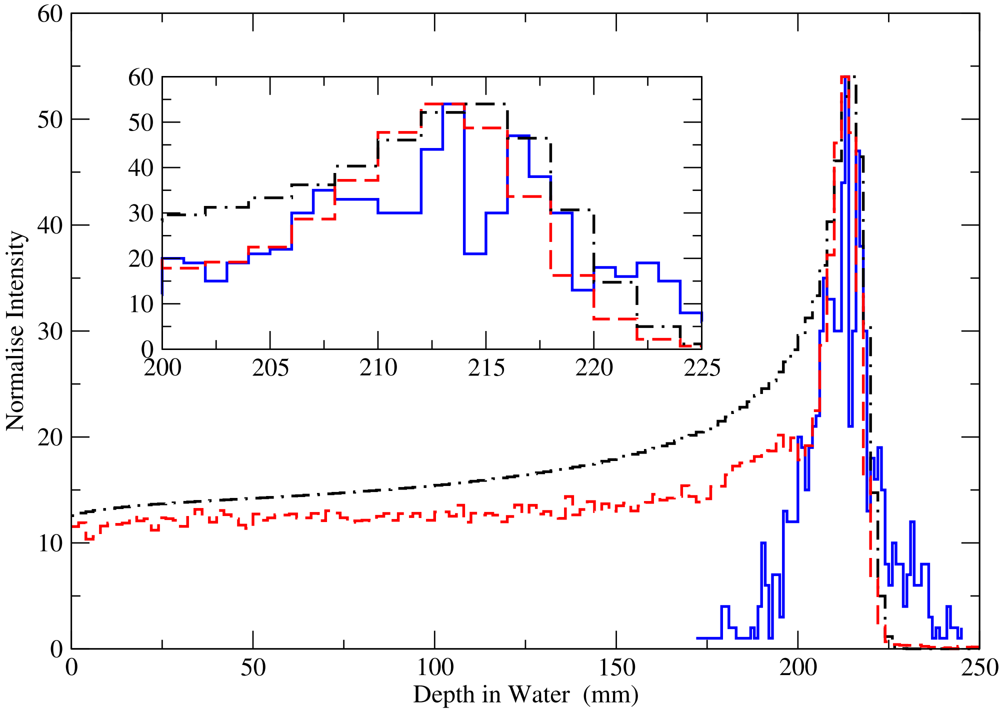

Figure S2. A clinical 180 MeV proton pencil beam impinges a water phantom. Two quantities are scored by the phantom: the proton energy deposition (*black* curve) and the 2.741 & 6.128 MeV ^{16}O-induced PG-ray (*red* curve). In addition addition the maximum intensity emission origin of the 2.741 & 6.128 MeV ^{16}O-induced PG-ray, detected with the spectrometer and reconstructed with the algorithm, is plotted (*blue* curve). Distributions are shown along the Z axis. The internal radius of the spectrometer is: 25 cm (solid angle Ω = 3% subtended by the spectrometer at the central point (0, 0, 0)).
